# A novel method for landslide displacement prediction by integrating advanced computational intelligence algorithms

**DOI:** 10.1038/s41598-018-25567-6

**Published:** 2018-05-08

**Authors:** Chao Zhou, Kunlong Yin, Ying Cao, Bayes Ahmed, Xiaolin Fu

**Affiliations:** 1 Engineering Faculty, China University of Geosciences, Wuhan, 430074 China; 2 Institute for Risk and Disaster Reduction, University College London (UCL), London, WC1E 6BT UK; 3 Administration of Prevention and Control of GeoHazards in the Three Gorges Reservoir of China, Yichang, 443000 China

## Abstract

Landslide displacement prediction is considered as an essential component for developing early warning systems. The modelling of conventional forecast methods requires enormous monitoring data that limit its application. To conduct accurate displacement prediction with limited data, a novel method is proposed and applied by integrating three computational intelligence algorithms namely: the wavelet transform (WT), the artificial bees colony (ABC), and the kernel-based extreme learning machine (KELM). At first, the total displacement was decomposed into several sub-sequences with different frequencies using the WT. Next each sub-sequence was predicted separately by the KELM whose parameters were optimized by the ABC. Finally the predicted total displacement was obtained by adding all the predicted sub-sequences. The Shuping landslide in the Three Gorges Reservoir area in China was taken as a case study. The performance of the new method was compared with the WT-ELM, ABC-KELM, ELM, and the support vector machine (SVM) methods. Results show that the prediction accuracy can be improved by decomposing the total displacement into sub-sequences with various frequencies and by predicting them separately. The ABC-KELM algorithm shows the highest prediction capacity followed by the ELM and SVM. Overall, the proposed method achieved excellent performance both in terms of accuracy and stability.

## Introduction

Landslides are a common natural hazard and cause extensive losses in mountainous areas. For example, the Maoxian landslide that occurred in the southwest of China in June 2017, damaged 62 houses and more than 100 people were buried^1–3^. In the north of Vietnam, a massive amount of soil and rocks, triggered by heavy rainfall, rolled down in an adjacent hill and caused 8 fatalities in October 2017^4^. In order to reduce such landslide disaster risks, developing an effective early warning system (EWS) should be prioritized in landslide research^5,6^.

Landslide displacement prediction is considered as an essential component for developing EWSs. It can be used to set warning thresholds and to recognize when a landslide undergoes an unpredicted acceleration^6,7^. Since Saito (1965) proposed the empirical formula for landslide prediction^8^, numerous landslide prediction models have been developed^9–16^. They can be grouped into two categories: physical-based models and data-based models. Physical-based models provide a good understanding and prediction of landslide failure. Jiang *et al*. (2011) investigated and predicted landslide deformation by establishing a three-dimensional geological model and performing a numerical simulation with the saturated–unsaturated fluid–solid coupling theory^17^. The physical-based models are complex and time consuming^18,19^, as they need to consider many conditions, such as complex landslide geometries, spatial variations in soil properties and three-dimensional groundwater flow etc.^19^. The data-based models have been applied recently because of their good prediction accuracy and simplicity. Du *et al*. (2012) analyzed the deformation characteristics of colluvial landslides and conducted the displacement prediction using a Back Propagation Neural Network (BPNN) method^20^. Zhou *et al*. (2016) integrated time series decomposition and support vector machine (SVM) to establish a displacement prediction method by considering the response relationship between triggering factors and landslide deformation^21^.

Although these methods perform well and provide crucial parameters for EWS, they require a huge amount of monitoring data to analyze the deformation mechanism. The least developed landslide-prone regions suffer the most^22^ because they cannot afford or do not have the available resources to monitor the parameters for landslide deformation analysis and prediction, such as information about groundwater, shear tension, pore water pressure etc. To overcome the limitations, a variety of computational intelligence methods have been applied in landslide study to achieve scientifically valid and accurate results^23–28^. These methods can maximize the extraction of useful information from limited data for landslide displacement prediction.

Landslide evolution is a complex nonlinear dynamic process, and is influenced by geological conditions of landslide mass and external triggering factors (e.g. precipitation, earthquake, human engineering activities, etc.). The displacement data includes multiple deformation information with various rules^29^. Wavelet transform (WT) is an effective signal processing algorithm which can decompose time series signals into several components with different frequencies. Many researchers have tried to predict landslide using artificial neural networks (ANN) methods^30–34^. The conventional ANN models can process nonlinear problems, but the selection of network structure and parameters is mainly dependent on experience. In addition, some other drawbacks of the traditional ANNs models are associated with excessive tunable parameters, slow learning rate, high possibility of entrapment in local minima, long computational time, and over-tuning^35^. Extreme learning machine is an algorithm based on single-hidden layer feed-forward neural network, which has been reported with good prediction capacity^36–40^. However, the random connection weights between the input and hidden layers make the output of ELM fluctuant. Consequently, ELM was introduced into Kernel learning and the kernel-based ELM (KELM) was proposed by Huang *et al*.^41^. Considering that the performance of KELM is affected by its parameters^38,41^, it is significant to select appropriate parameters. The artificial bee colony (ABC) is a swarm intelligence-based global optimization algorithm, and has been utilized to search the optimal parameters of KELM due to its high accuracy and fast convergence characteristic^42^.

In this study, the advanced computational intelligence algorithms namely the WT, ABC and the KELM methods (WT-ABC-KELM) were integrated to establish a novel landslide displacement prediction method. The Shuping landslide in the Three Gorges Reservoir area (TGRA) of China was undertaken as a case study. The ABC-KELM, WT-ELM, ELM, and SVM methods were applied for performance comparison.

## Case study

### Geological conditions

**Figure 1:**
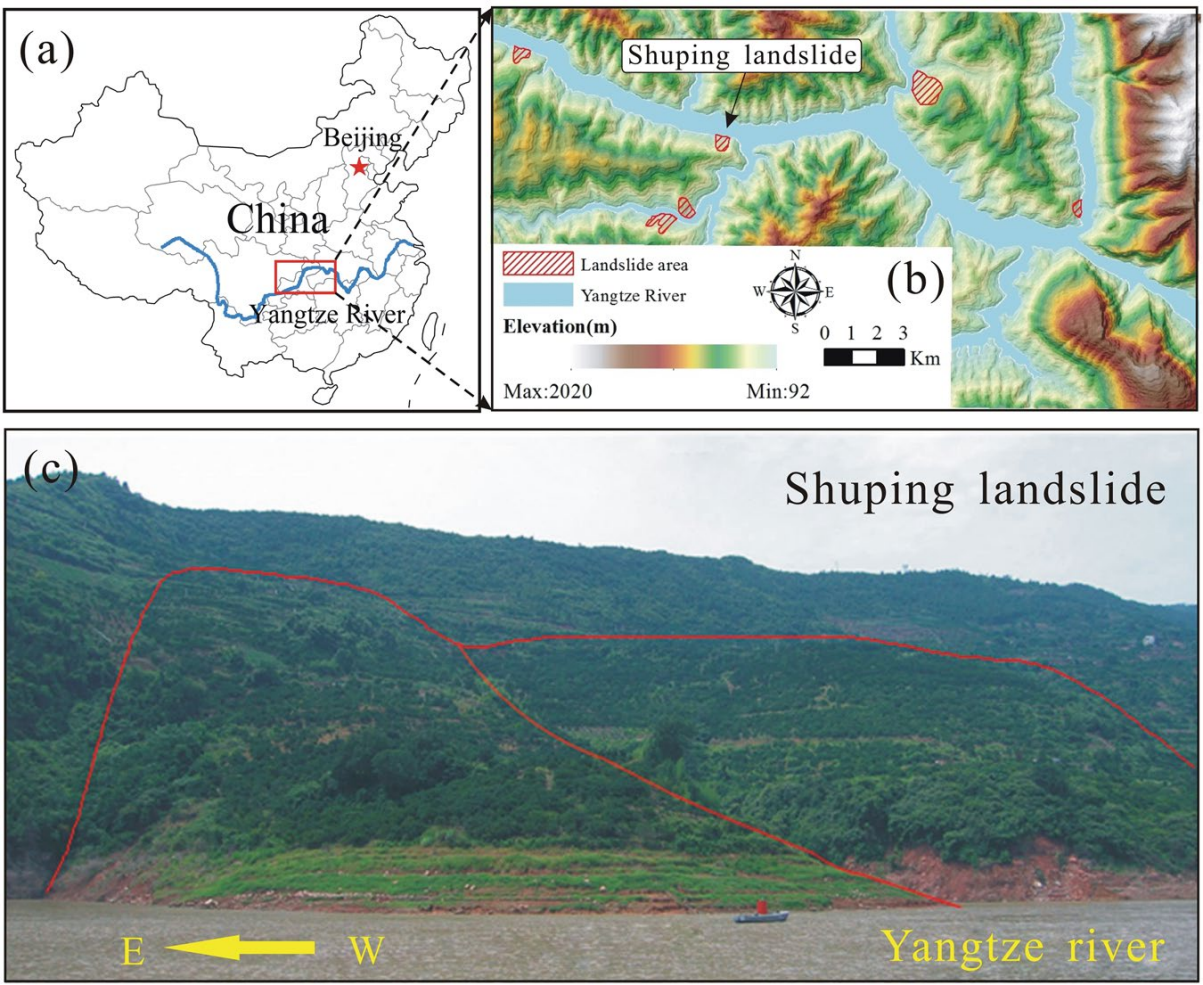
(**a**) Map of China; (**b**) Map showing the location of the Shuping landslide; and (**c**) Photograph of the Shuping landslide (The two maps was created by Chao Zhou using ArcGIS 9.3, http://www.esri.com/).

**Figure 2:**
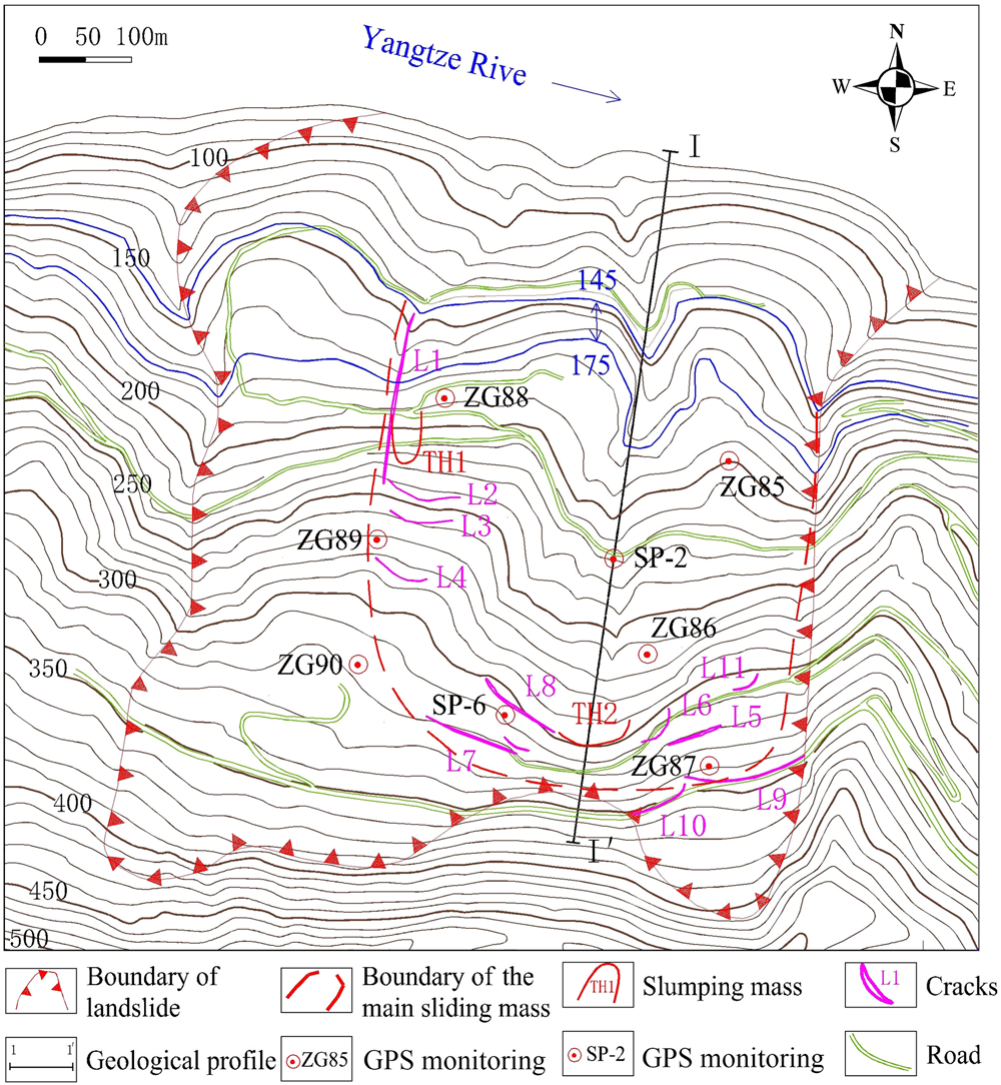
Topographical map of the Shuping landslide, with the location of the monitoring networks (Drawn by Ying Cao using AutoCAD 2014, https://www.autodesk.com.cn/), the cross-section can be seen in Fig. 3.

**Figure 3:**
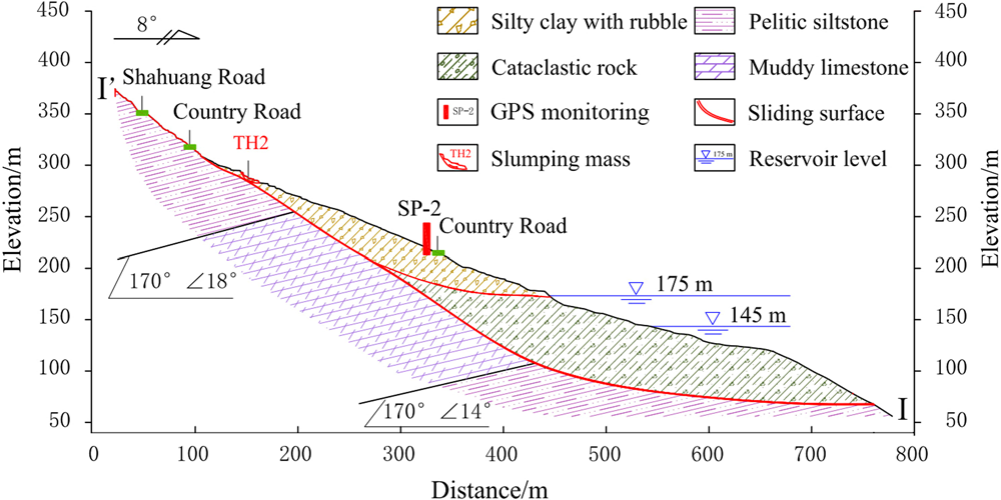
Schematic geological cross-section (I-I’) of the Shuping landslide (Drawn by Ying Cao using AutoCAD 2014, https://www.autodesk.com.cn/), the track can be seen in Fig. 2.

The Shuping landslide occurred in the Zigui county that is located in the TGRA (30°59′37″N, 110°37′0″E). The landslide location is near the Yangtze River and 47 kilometers from the Three Gorges Dam (Fig. 1). The landslide is fan-shaped in plane with a main sliding direction of N11°E. The left and right boundaries are defined by two gullies, while the elevation of the upper boundary varies between 340 m and 400 m a.s.l., and the elevation of the toe is 60 m a.s.l. The landslide covers an area of 5.5 × 10^5^ m^2^, with a mean longitudinal dimension of 800 m and a width of 700 m (Fig. 2). The Shuping landslide is an ancient slumping deposit. It developed in the reverse syncline slope which is composed of mudstone and siltstone with muddy limestone of Triassic Badong Formation, with the dip direction of 120~173° and the dip angle of 9~38°. The mean depth of the sliding surface is about 50 m (Fig. 3). The Shuping landslide is a large colluvial mass movement with an estimated volume of 2750 × 10^4^ m^3^. The eastern and middle part of the landslide is the main sliding mass with greater deformation. The area and volume of which are 35 × 10^4^ m^2^ and 1575 × 10^4^ m^3^, respectively.

### Deformation characteristics

The Shuping landslide is an ancient landslide with complex geological conditions and a large volume. The deformation was significantly slow before the construction of the Three Gorges Dam (see boundary of landslide in Fig. 2). After the construction of the dam, the water level of Yangtze River raised from 75 m to 145 m a.s.l. Under the influence of such periodic reservoir fluctuations and heavy precipitation, the Shuping landslide revived. The landslide deformed partially which is shown as the main sliding mass in Fig. 2. The deformation monitoring of the sliding body is mainly based on the global positioning system (GPS) and macroscopic geological inspection. Eight effective GPS monitoring stations were installed surrounding the Shuping landslide (ZG85-ZG90, SP-2 and SP-6) (Fig. 2), with the layout of two vertical and three horizontal directions to monitor the deformation of the whole landslide.

**Figure 4:**
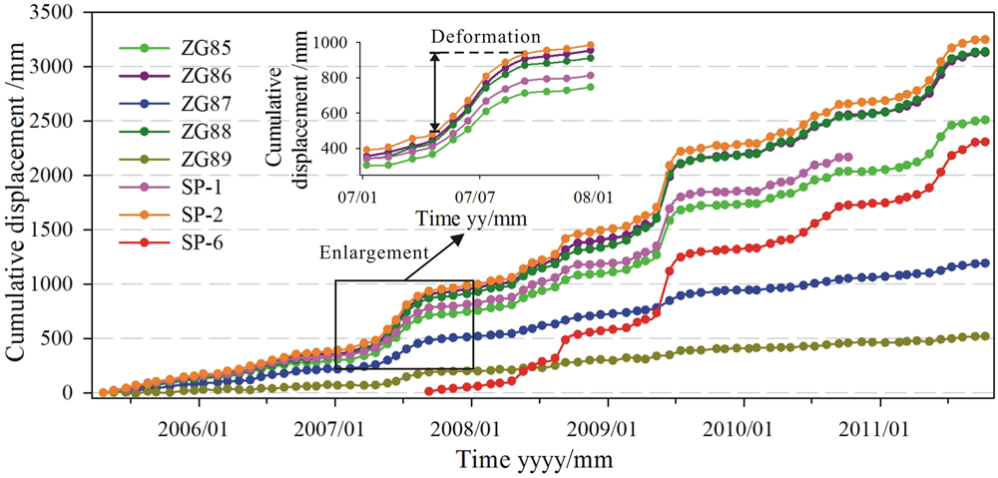
The GPS monitoring data of the Shuping landslide.

Under the influence of periodic reservoir fluctuations and seasonal precipitations, the displacement speed of the Shuping landslide is found to be increasing between May and September in 2007 (Fig. 4). In this period, the landslide deformed 453.5 mm while the total deformation of 2007 was 593 mm. Several transverse arc-shaped tensile cracks occurred on the eastern upper part of the landslide (L5-L11), with the length of about 20 m and the width of 5~8 m. From June 13 to July 11 in 2007, the landslide experienced severe deformation. The maximum deformation of SP-2 and ZG86 in the middle part was about 130 mm (Fig. 4), and the maximum daily speed reached around 5.10 mm.

**Figure 5:**
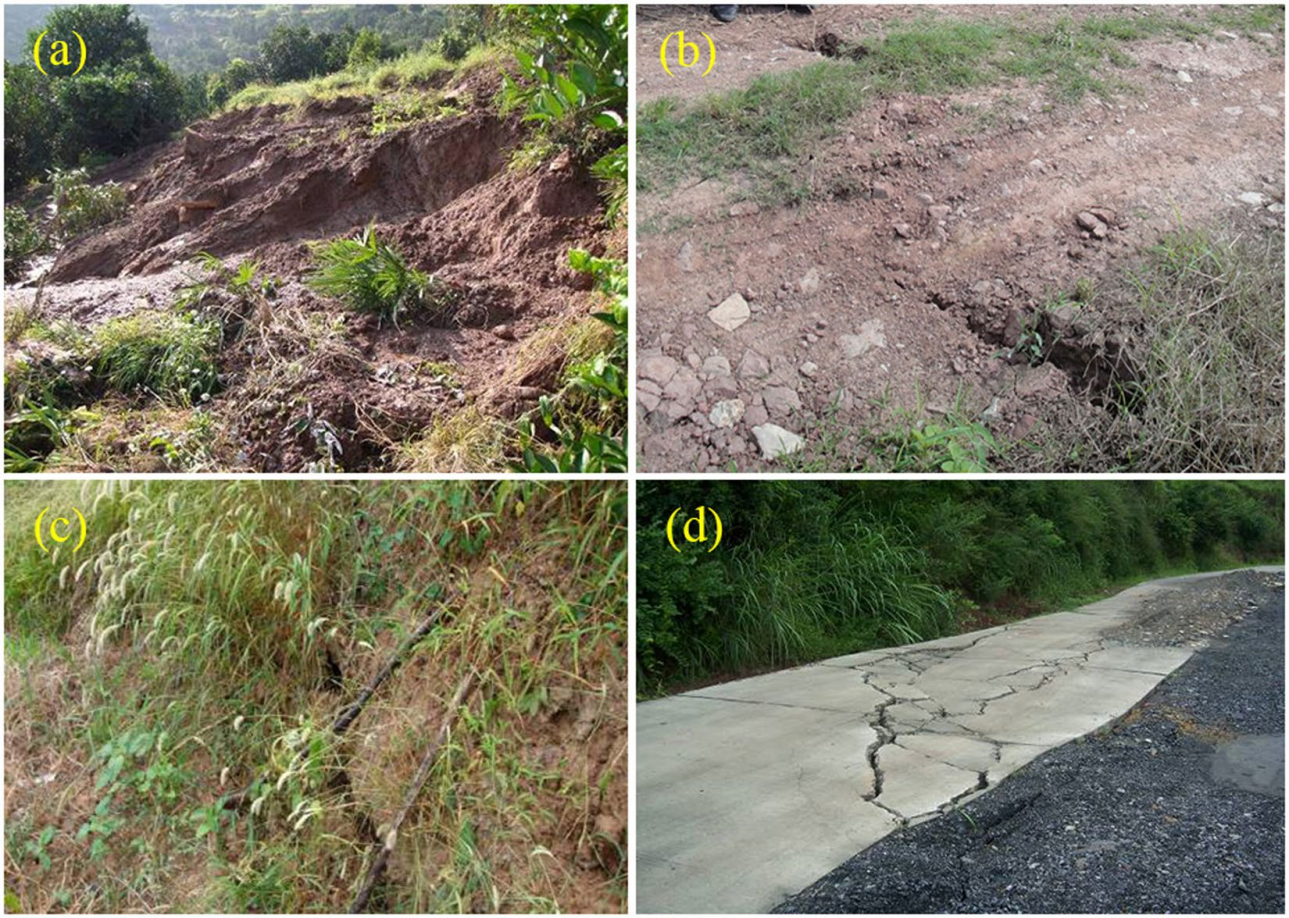
The deformation phenomena on the landslide mass: (**a**) slump of TH1, (**b**) crack of L2,(**c**) crack of L4, and (**d**) crack of L9.

Due to heavy rainfall in the late August 2008, the cracks on the eastern part showed further development, and were connected with the cracks of L9 and L10. The direction of all the cracks was basically consistent with the upper landslide boundary (Fig. 2). Meanwhile, a small-scale slump (TH1, Fig. 5a) occurred in the west of the landslide with a length and width of 50 m and 30 m respectively, and a volume larger than 1000 m^3^. In August 2008, the GPS monitoring stations of SP-2 in the middle part and SP-6 in the upper part showed larger deformation than the other monitoring stations. Their monthly deformation was found 143.7 mm and 169.1 mm respectively.

The deformation speed of the Shuping landslide severely increased between May and August in 2009. The most serious deformation occurred in June with crack extension and road subsidence on the upper landslide (L2 -L4) (Fig. 5b,c). All the cracks on the eastern part and most of the echelon cracks on the western part were connected. The displacements monitored by the GPS stations (GPS85, GPS86, GPS88, SP-2, SP-6) were over 300 mm within a month (Fig. 4). Probably it formed the boundaries of the main sliding zone of the Shuping landslide.

During the year of 2010 and 2011, the deformation of the main sliding mass increased since May, and more surface cracks developed and increased into larger scales on the boundaries. The cracks on the eastern boundary were of compression-shearing character (L11), the cracks on the upper boundary were formed by tensile force (L9 and L10) (Fig. 5d), and the cracks on the western boundary are of shearing-tension character with echelon distribution (L2 -L4) (Fig. 2).

## Results and Discussion

### The decomposition of landslide displacement

**Figure 6:**
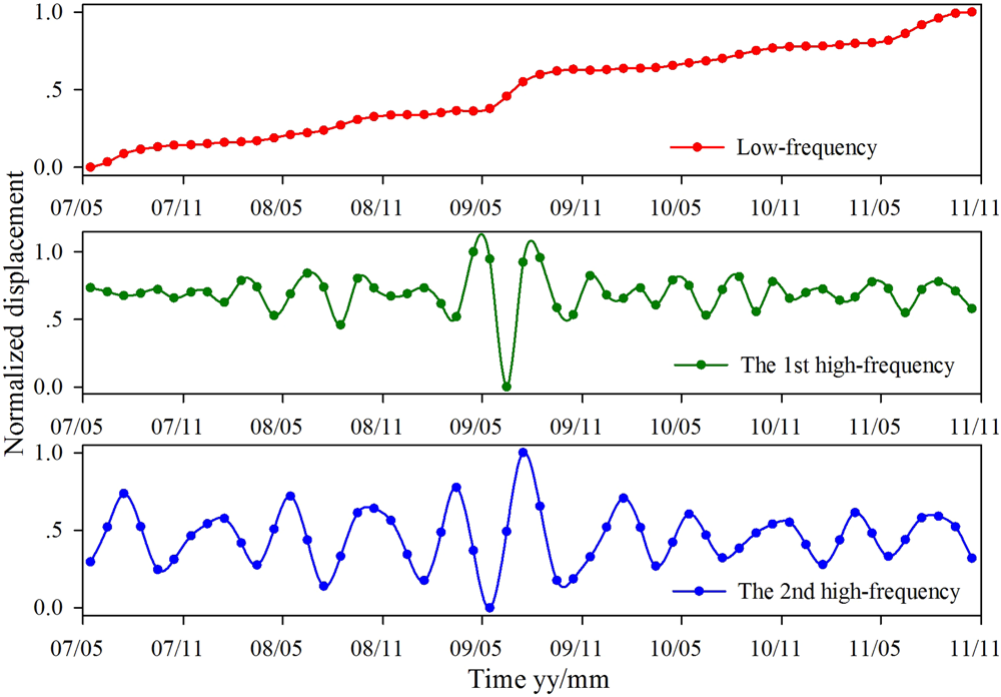
The decomposition result of the total displacement of SP-2.

Landslide total displacement can be decomposed into multi-level sub-sequences with various frequencies using the WT. In order to select an appropriate number of decomposition levels, Equation (1)^43–47^ was used in the proposed method:

1 $$L=\,{\rm{int}}[\mathrm{log}(N)]$$

where *L* is the decomposition level, and *N* is the number of time series data. Here, *N* is 54. The total displacement sequence was decomposed into 2 levels (i.e. *L*), which can be expressed as follows:

2 $${D}_{s}={D}_{a}+{D}_{h1}+{D}_{h2}$$

where *D*_*s*_ is the original total displacement; *D*_*a*_ is the low-frequency component; and *D*_*h*1_ and *D*_*h*2_ are the first and second high-frequency components, respectively. As the computational intelligence algorithms are more sensitive in their desired range, each sub-sequence of displacement was normalized into the desired range between [0,1] based on Equation (3) before modelling.

3 $${D}_{i}=\frac{{D}_{ori,i}-{D}_{ori,min}}{{D}_{ori,max}-{D}_{ori,min}}$$

where *D*_*i*_ are the normalized values, *D*_*ori,i*_ are the original values, ${D}_{ori,min}$ is the minimum value of an original sub-sequence, and ${D}_{ori,max}$ is the maximum value of an original sub-sequence. As shown in Fig. 4, the GPS station of SP-2 experienced the largest deformation. In this study, the displacement data of SP-2 form May 2007 to October 2010 was used for model training, and the data after October 2010 was used for performance test. After data normalization, the decomposition results of SP-2 are shown in Fig. 6.

### The modelling of displacement prediction

In the modelling of the proposed method, the radial basis function was adopted as the kernel function of KELM, and the parameters of the KELM, i.e. regularization coefficient *c* and kernel parameter *γ*, were optimized through the ABC algorithm and the training process. In the hybrid model of ABC-KELM, the parameters of *c* and *γ* were set as solution target, the KELM was set as solution function, and the root mean square error (REMS) of the fitting result of KELM was set as fitness (best cost). In the optimization processing, the problem solution (*c* and *γ*) was selected through the strategy of ABC algorithm, and the corresponding REMS was calculated for fitness comparison (see the Methods Section). The initial parameters of the ABC algorithm, namely the maximum number of iterations, the number of population, and the acceleration threshold were set as 30, 50, and 20, respectively.

**Figure 7:**
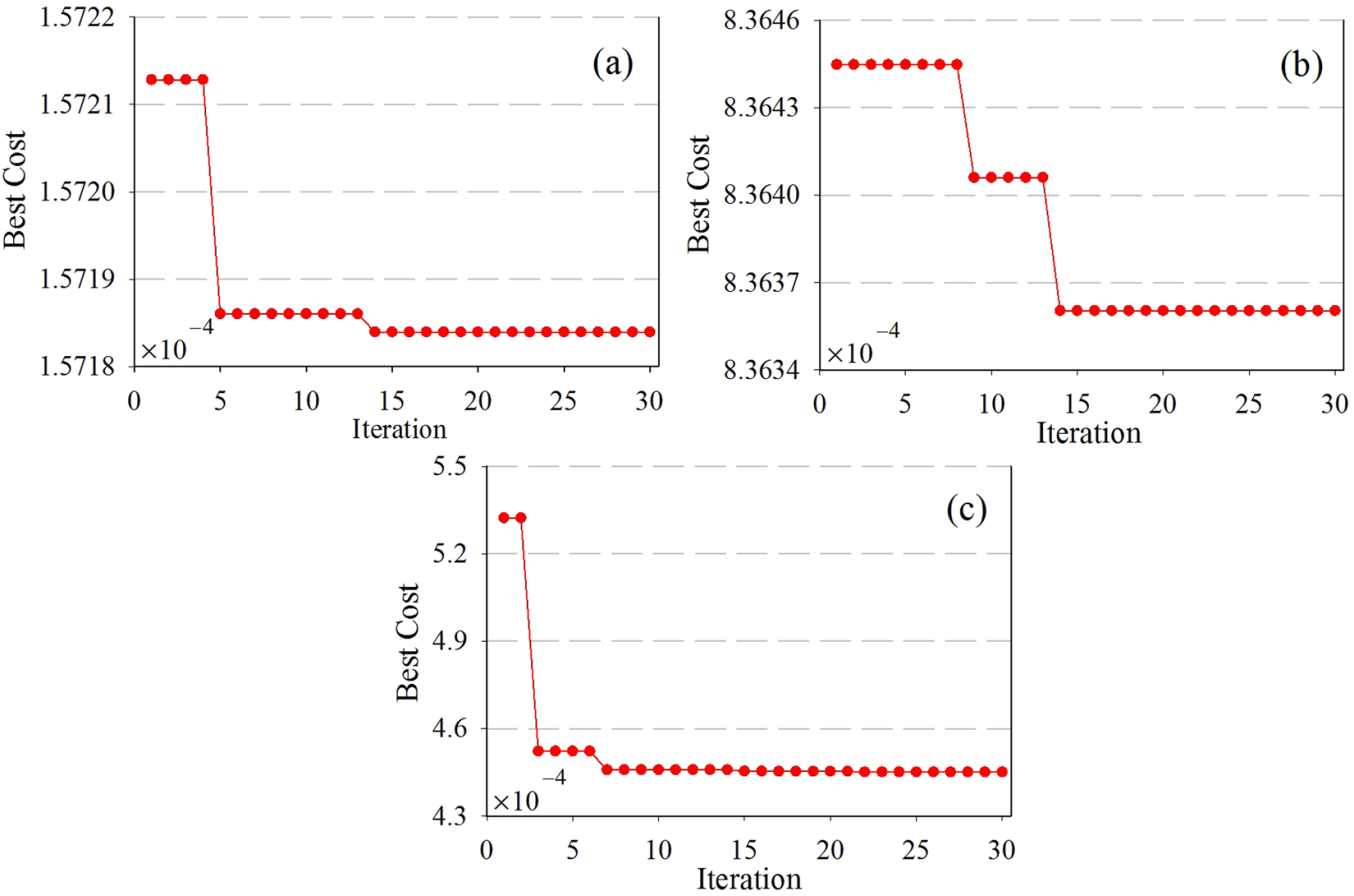
The parameters searching process for the three sub-sequences predictions: (**a**) the low-frequency, (**b**) the 1st high-frequency, and (**c**) the 2nd high-frequency.

**Figure 8:**
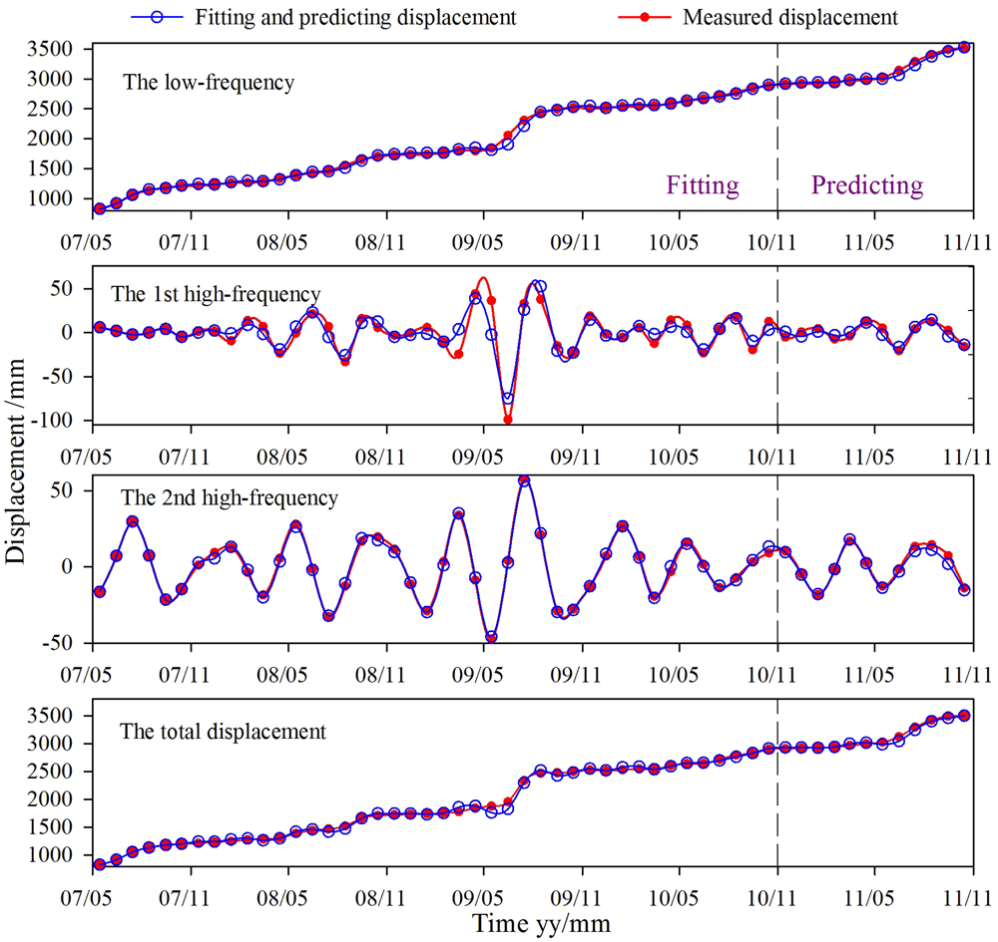
The results of fitting and prediction values of SP-2.

Each displacement sub-sequence was predicted separately using the ABC-KELM model. The best cost in the parameters optimization processes of the three sub-sequences is shown in Fig. 7. The optimized parameters and inputs for each sub-sequence modelling are shown in Table 1. The fitting and prediction results of each sub-sequence can be calculated (Fig. 8) based on Equation (3). As per the decomposition principle of the displacement time series (Equation (2)), the predicted total displacement was obtained by summing up the predicted values of all the sub-sequences (Fig. 8).

**Table 1:** The parameters and inputs of the prediction models for sub-sequences.

Sub-sequences	Parameters	Inputs
The low-frequency	$c=1.0\ast {10}^{6},\gamma =1727.0881$	_[*d (t-3)*, *d (t-2)*, *d (t-1)*]_
The 1st high-frequency	$c=3.35\ast {10}^{4},\gamma =929.1308$	_[*d (t-6)*, *d (t-5)*, *d (t-4)*, *d (t-3)*, *d (t-2)*, *d (t-1)*]_
The 2nd high-frequency	$c=1.0\ast {10}^{6},\gamma =38.1863$	_[*d (t-6)*, *d (t-5)*, *d (t-4)*, *d (t-3)*, *d (t-2)*, *d (t-1)*]_

Note: *d(t-n)* means the displacement of *n* months ago.

**Figure 9:**
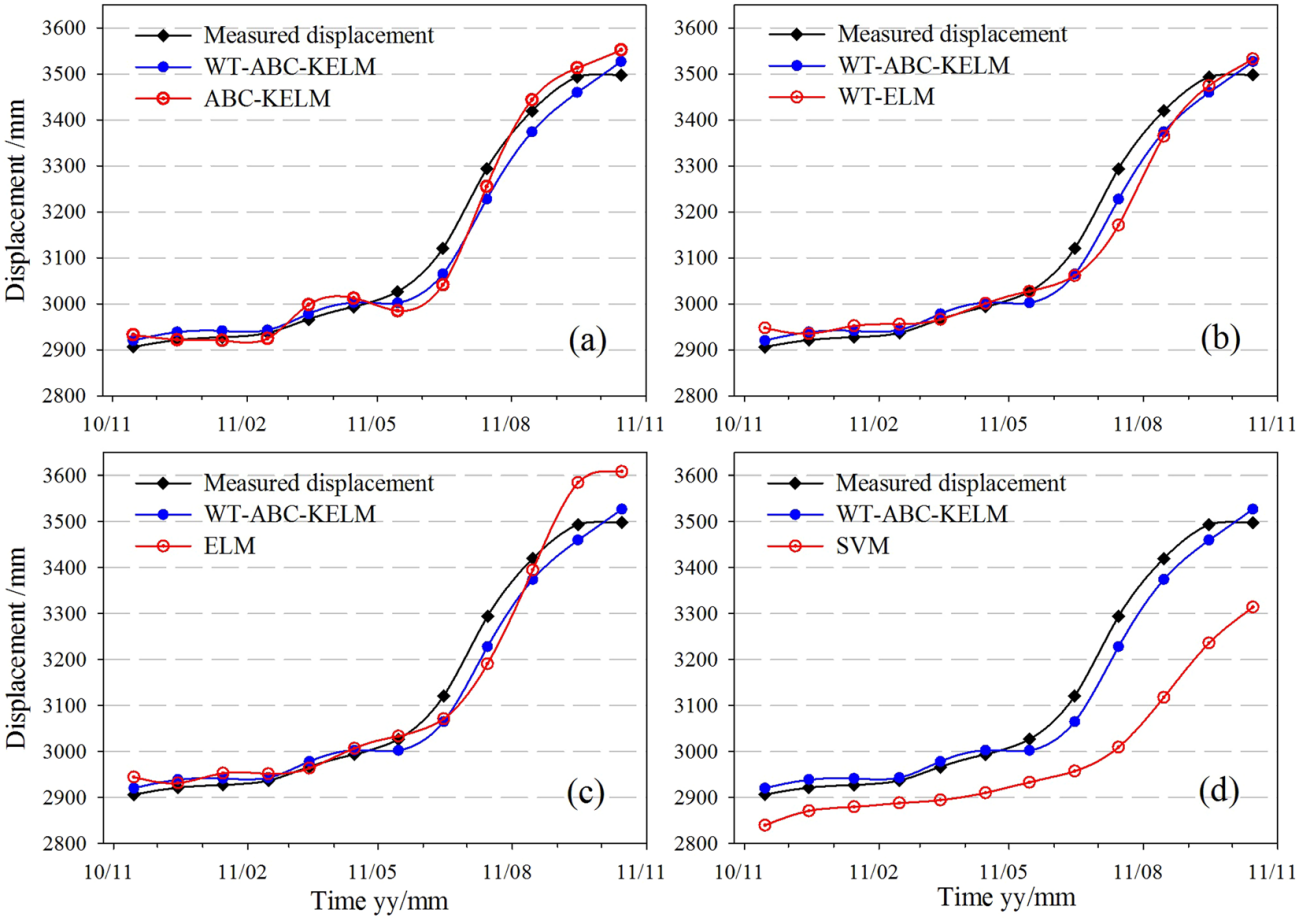
The predicted results of the compared methods: (**a**) ABC-KELM, (**b**) WT-ELM,(**c**) ELM, and (**d**) SVM.

To verify the performance of the proposed method, four methods, namely, the ABC-KELM, WT-ELM, ELM and SVM^21,48^, were executed with the same data. Regarding the ABC-KELM method, the parameters of KELM were optimized using the ABC algorithm. The optimal regularization coefficient and the kernel parameter were 1.0*10^6^ and 1492.9397, respectively. The number of the neurons in the hidden layer (i.e. an integer) is the sole parameter of the ELM algorithm. It was selected by the trial-and-error approach within the range from 1 to 50. For the WT-ELM method, the number of the neurons of the low-frequency, the 1st high- frequency, and the 2nd high-frequency were 10, 15, and 21, respectively. For the ELM method, the number of the neurons was set as 10. The radial basis function was applied as the kernel function of SVM, and the penalty factor ɵ and the kernel function g were set as 85.4376 and 0.01, respectively. The parameters and inputs of the four models used for comparison are shown in Table 2. Their predicted total displacements are shown in Fig. 9.

**Table 2:** The parameters and inputs of the four compared methods.

Methods	Parameters	Inputs
_ABC-KELM_	$c=1.0\ast {10}^{6},\gamma =1492.9397$	_[*d (t-2)*, *d (t-1)*]_
_ELM_	*n* = 10	_[*d (t-2)*, *d (t-1)*]_
_SVM_	Θ = 85.4376, *g* = 0.01	_[*d (t-2)*, *d (t-1)*]_
_WT-ELM_		
_The low-frequency_	*n* = 10	_[*d (t-3)*, *d (t-2)*, *d (t-1)*]_
_The 1st high-frequency_	*n* = 15	_[*d (t-6)*, *d (t-5)*, *d (t-4)*, *d (t-3)*, *d (t-2)*, *d (t-1)*]_
_The 2nd high-frequency_	*n* = 21	_[*d (t-6)*, *d (t-5)*, *d (t-4)*, *d (t-3)*, *d (t-2)*, *d (t-1)*]_

### Accuracy comparison and discussion

Validation is an essential component in testing the effectiveness and scientific significance of the method applied for landslide displacement prediction study. Three statistical indexes –the root mean square error (RMSE), the mean absolute percentage error (MAPE), and the correlation coefficient (R)– were used for assessing the prediction accuracy of the five prediction methods (Table 3).

**Table 3:** The accuracy comparison of the prediction methods.

Methods	REMS	MAPE	R
WA-ABC-KELM	33.13	0.85%	0.991
WA- ELM	36.23	0.93%	0.989
ABC-KELM	46.79	1.04%	0.980
ELM	53.00	1.25%	0.977
SVM	166.39	4.25%	0.959

As shown in Fig. 8, the proposed method i.e. WT-ABC-KELM achieved high accuracy both in model fitting and prediction stages. To compare the performance of the ABC-KELM and ELM algorithms, the accuracy of the WT-ABC-KELM and ABC-KELM methods were compared with the WT-ELM and ELM, respectively. The accuracy statistic parameters (REMS, MAPE and R) of the WT-ABC-KELM are calculated 33.13, 0.85%, and 0.991, respectively (Table 3), which are found better than the WT-ELM. The REMS, MAPE and R of the ABC-KELM are estimated 46.79, 1.04%, and 0.980, respectively. It indicates the ABC-KELM algorithm can simulate the actual situation more accurately than the ELM. The predicted values of the ABC-KELM algorithm are closer to the measured values. The REMS, MAPE and R of the SVM are calculated 166.39, 4.25%, and 0.959, which indicates the prediction capacity of the SVM algorithm is worse than the ABC-KELM and ELM algorithms. It can be stated that the ABC-KELM algorithm has the strongest prediction capacity followed by the algorithm of ELM and SVM.

As reported in the previous literature^38,41,49,50^, the ELM outperforms the conventional computational intelligence algorithms. However, the connection weights between the input and hidden layers of the ELM algorithm are randomly determined and it leads to random fluctuation of the predicted results. In KELM algorithm, a fixed kernel function is applied to replace the output function to avoid the randomly generating process of the connection weights in the ELM algorithm. Under the same condition (Table 2), ten trials were performed using the ABC-KELM and ELM methods, and the REMS values of predicted results are shown in Table 4. Apparently, the predicted results of the ELM method are fluctuant, and the RMSE variance of the 10 predictions is as high as 184.12. On the contrary, the predicted results of the ABC-KELM method in different trials are the same without fluctuations. For generating landslide early warnings, the fluctuations of the predicted results as found in the case of ELM may produce false warnings and mislead disaster managers into making erroneous decisions. Overall, the ABC-KELM outperforms the ELM slightly. The prediction of the KELM is fixed and that is crucial in practice.

**Table 4:** The comparison of the REMS values between the ABC-KELM and ELM in 10 trial.

Model	1st	2nd	3rd	4th	5th	6th	7th	8th	9th	10th	Mean	Variance
ABC-KELM	46.79	46.79	46.79	46.79	46.79	46.79	46.79	46.79	46.79	46.79	46.79	0
ELM	53.00	63.76	76.72	61.72	78.12	63.15	96.65	60.11	90.04	81.84	72.51	184.12

Landslide deformation is a complex nonlinear process affected by many factors and shows highly nonlinear total cumulative displacement sequence. The WT can decompose the total displacement into sub-sequences with various frequencies for easier prediction. As shown in Table 3, the REMS, MAPE and R of the ABC-KELM are calculated 46.79, 1.04%, and 0.980, respectively. Meanwhile, the three statistic indexes of the ELM are 53.00, 1.25%, and 0.977, respectively. Notably, the prediction accuracy of both methods is found lower than the corresponding hybrid methods using the WT (Table 3 and Fig. 9). The results of performance comparison demonstrate that decomposing the total displacement into several sub-sequences and predicting them separately can effectively improve the prediction accuracy.

The computational intelligence methods can accurately predict landslide displacement as demonstrated in the current case study (Table 3 and Fig. 9). The ANN algorithms, including the KELM, require past data for training. The past data is the foundation for forecasting and the prediction accuracy depends on the training data (past displacement). When the deformation pattern in prediction period is similar to the training period, for example, the two periods are under the same evolution stage, the landslide displacement can usually be predicted accurately. Conversely, when the deformation pattern in predicting period is different from the training period, the performance of these training-based models will be limited. It is important to select the monitoring data as training samples whose deformation pattern is similar to the prediction period.

## Conclusions

The conventional methods for landslide displacement prediction require enormous professional monitoring data. To overcome such limitations, a novel method is proposed by integrating the WT, ABC and KELM algorithms (WT-ABC-KELM). The Shuping landslide in the TGRA in China is taken as a case study. The WT-ELM, ABC-KELM, ELM, and SVM methods were implemented for comparison purpose. The results suggest:

- The prediction methods applying the WT algorithm performed better. The WT algorithm can decompose a highly nonlinear series of landslide displacement into sub-sequences with different frequencies to reduce the difficulty of prediction and improve the prediction accuracy.
- The ABC algorithm can efficiently optimize the parameters of the KELM, and the hybrid model of ABC-KELM outperformed the ELM and SVM models. The predicted value of KELM is fixed compared to the ELM algorithm.

Overall, the proposed WT-ABC-KELM method integrates the advantages of the three computational intelligence algorithms and produces better prediction accuracy than the WT-ELM, ABC-KELM, ELM, and SVM methods.

## Methods

### Wavelet transform

The wavelet transform (WT) is an effective algorithm in signal processing, which provides good localization features in both time and frequency domains. The original signal can be decomposed by a set of wavelet functions and are obtained by stretching and translating the mother wavelet. The mother wavelet can be defined as follows:

4 $$\{\begin{array}{c}{\int }_{-\infty }^{+\infty }\varnothing (t)=0\,\\ {\varnothing }_{a,b}(t)={|a|}^{-0.5}\varnothing (\frac{t-b}{a})\end{array}\,$$

where ${\varnothing }_{a,b}(t)$ is a successive wavelet function, *a* is a frequency factor, *b* is a time factor. The WT algorithms can be divided into two classes: the continuous wavelet transformation (CWT) and discrete wavelet transformation (DWT). Compared to the CWT, which requires complex computation and massive data, the DWT requires less time and is easy to implement.

The DWT algorithm proposed by Mallat (1989)^51^ has been widely used. It applies high-pass and low-pass filters to extracting approximation and detail sequence from the original signal. From a given signal *S(t)*, one approximation series *a* and *n* detail series (*d*_1_*,…, d*_*n*_) can be extracted by Mallat’s algorithm. There are many choices for the wavelet functions, such as the Haar^52^, Meyer^53^, Daubechies^54^ and so on. The Daubechies wavelet (db *N*, where *N* is the degree of wavelet) is smooth, orthogonal and compactly supported. In the proposed method, the wavelet function of db 4 was utilized to decompose landslide displacement sequence.

### Kernel-based extreme learning machine

The extreme learning machine (ELM), proposed by Huang *et al*. (2006), is a single-hidden layer feed-forward neural network (SLFN) with randomly generated hidden nodes^36^. Because of its excellent generalization ability and fast learning speed, the ELM has been adopted in various fields recently^55–57^. For *N* arbitrary samples $({x}_{i},{t}_{i})$, where ${x}_{i}={[{x}_{i1},{x}_{i2},\cdots ,{x}_{im}]}^{T}\in {R}^{m}$, ${t}_{i}={[{t}_{i1},{t}_{i2},\cdots ,{t}_{im}]}^{T}\in {R}^{n}$, the standard SLFNs with *M* hidden nodes and activation function g(*x*) are mathematically written as:

5 $$\sum _{i=1}^{M}{\beta }_{i}{\rm{g}}({W}_{i}\cdot {X}_{j}+{b}_{i})={O}_{j},j=1,2,\cdots ,N\,$$

where ${W}_{i}={[{W}_{1i},{W}_{2i},\cdots ,{W}_{mi}]}^{T}$ is the weight vector connecting the *i*th hidden node and the input nodes; ${\beta }_{i}={[{\beta }_{1i},{\beta }_{2i},\cdots ,{\beta }_{mi}]}^{T}$ is the weight vector connecting the *i*th hidden node and the output nodes; *b*_*i*_ is the threshold of the *i*th hidden node, ${W}_{i}\cdot {X}_{j}$ is the inner product of *W*_*i*_ and *X*_*j*_; and ${O}_{j}={[{O}_{j1},{O}_{j2},\cdots ,{O}_{jn}]}^{T}$. is the output vector of SLFNs.

The standard SLFNs with *M* hidden nodes and activation function g(*x*) can approximate these *N* samples with zero error, which means that $\sum _{j=1}^{M}\parallel {O}_{j}-{t}_{j}\parallel =0$, i.e., there exist *β*_*i*_, *w*_*i*_ and *b*_*i*_ such that

6 $$\sum _{i=1}^{M}{\beta }_{i}{\rm{g}}({w}_{i}\cdot {x}_{j}+{b}_{i})={y}_{j},j=1,2,\cdots ,N\,$$

The above equation can be expressed compactly as follows:

7 $$H\beta =Y\,$$

where *H* is the output matrix of the hidden layer. As the randomly selected input weight and the hidden-layer threshold are determined, the network training is equivalent to finding the least squares solution of $\hat{\beta }$. The equation can be written as:

8 $$\hat{\beta }={H}^{+}Y$$

where *H*^+^ is the Moore-Penrose generalized inversion of matrix *H*.

In order to overcome the randomness of the ELM algorithm, and to improve its generalization capability and stability, Huang *et al*. extended the ELM into kernel learning and proposed the kernel-based ELM (KELM)^39^. Based on the orthogonal projection method and ridge regression theory, the output weight *β* can be calculated by adding a positive constant *1/C* as:

9 $$\beta ={H}^{T}{(1/C+H{H}^{T})}^{-1}Y\,$$

Hence, the output function of ELM is shown as follows:

10 $$f(x)=h(x){H}^{T}{(1/C+H{H}^{T})}^{-1}Y$$

Usually, the output function *h*(*x*) is unknown for users, a kernel matrix for the ELM can be utilized to replace *h*(*x*). The output function of the KELM can be written as follows:

11 $$f(x)={[\begin{array}{c}K(x,{x}_{1})\\ \vdots \\ K(x,{x}_{N})\end{array}]}^{T}{(1/C+H{H}^{T})}^{-1}T$$

where *K*(*x*, *x*_*i*_) is the kernel function.

### Artificial Bee Colony algorithm

Artificial bee volony (ABC) is one of the latest optimization algorithms, and is being widely studied and successfully applied to multiple contexts^42^. The ABC algorithm was inspired by the intelligent foraging behaviour of honey bee swarms. In this algorithm, three types of bees are defined: the employed bees are associated with specific food sources (problem solutions), the onlooker bees in the hive watch the dance of employed bees and aim to choose a good food source (suboptimal solutions), and the scout bees search for new food sources (candidate solutions) in a random way.

**Figure 10:**
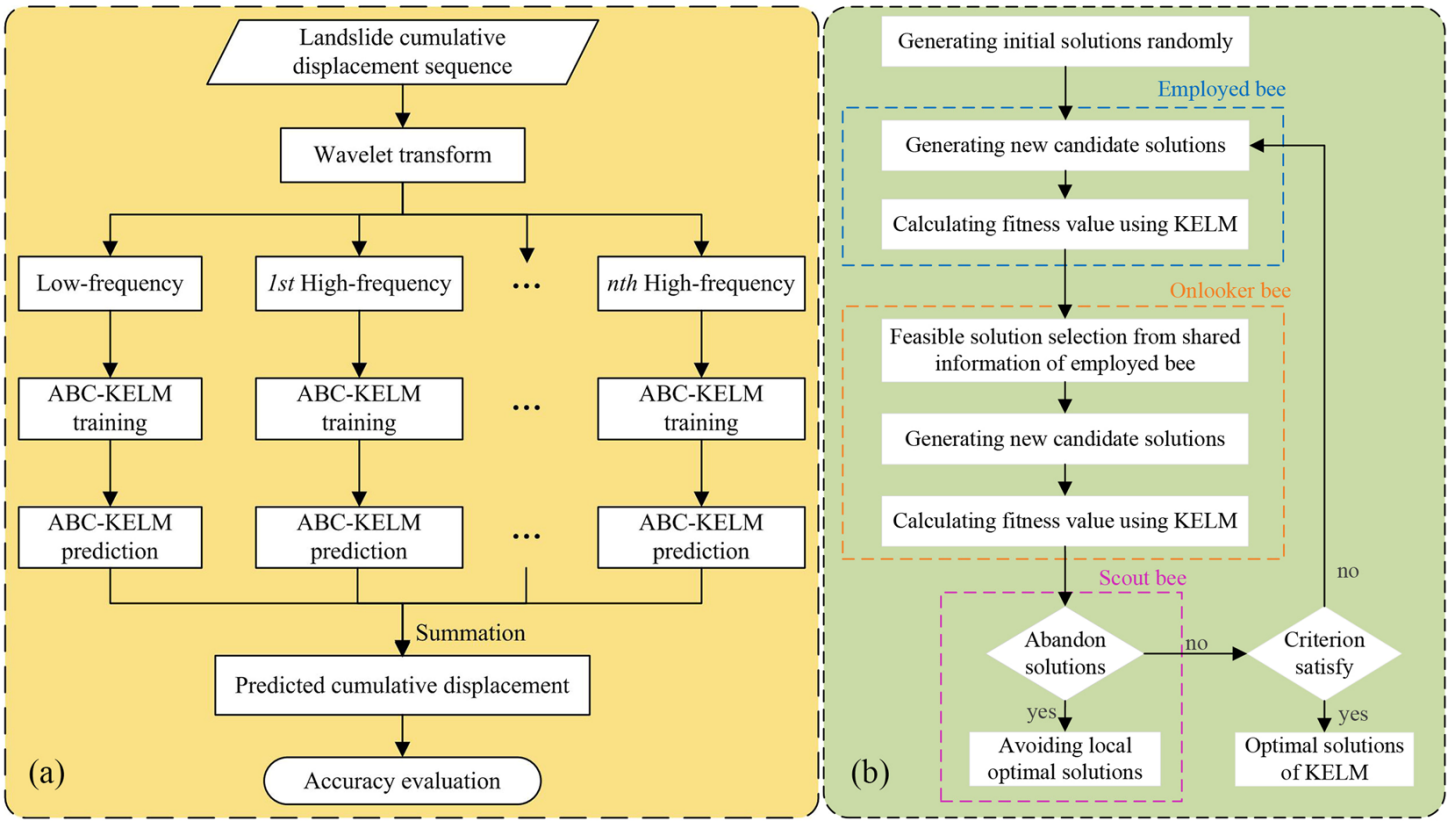
(**a**) The flowchart of the proposed forecast method; (**b**) the hybrid model of ABC-KELM.

The employed bees search for food sources in their memory and meanwhile they share the information of these food sources with the onlooker bees. The onlooker bees tend to select better food sources from those found by the employed bees. The food source with higher quality (lower best cost) will have a better chance to be selected by the onlooker bees than the one with lower quality. The scout bees are translated from a few employed bees, who abandon their food sources and search for new ones. In general, an iteration of ABC optimization algorithm contains four steps: the food source initialization, employed bee phase, onlooker bee phase, and the scout bee phase (Fig. 10b).

### The proposed coupling model and accuracy evaluation

Landslide displacement is caused by various factors and contains multi-level information of deformation. In the proposed method, the original total displacement is decomposed into several sub-sequences with different frequencies. Subsequently, each displacement sub-sequence is predicted separately by the KELM, while the parameters of KELM were optimized using the ABC. The predicted total displacement is obtained by summing up all the predicted displacement sub-sequences. The flowchart of the proposed prediction method is shown in Fig. 10.

Accuracy evaluation is an important component for performance test of the proposed model. The statistical indexes, such as the root mean square error (RMSE), mean absolute percentage error (MAPE) and correlation coefficient (R), were utilized to verify the efficiency of the proposed method. Smaller values of RMSE and MAPE, and larger value of R indicate higher prediction accuracy. The equations of the three indexes are shown as follows:

12 $$RMSE=\sqrt{\frac{1}{N}\sum _{i=1}^{N}{({\hat{D}}_{i}-{D}_{i})}^{2}}$$

13 $$MAPE=\frac{1}{N}\sum _{i=1}^{N}|\frac{{\hat{D}}_{i}-{D}_{i}}{{D}_{i}}|$$

14 $$R=\frac{{\sum }_{i=1}^{N}({D}_{i}-\bar{D})({\hat{D}}_{i}-\overline{\hat{D}})}{\sqrt{{\sum }_{i=1}^{N}{({D}_{i}-\bar{D})}^{2}}\sqrt{{\sum }_{i=1}^{N}{({\hat{D}}_{i}-\overline{\hat{D}})}^{2}}}$$

where *N* is the number of displacement values; *D*_*i*_ is the observed displacement value; ${\hat{D}}_{i}$ is the predicted displacement value; $\bar{D}$ is the mean of observed values; $\overline{\hat{D}}$ is the mean of predicted values.

### Data Availability Statement

The relevant datasets used in this study are available from the corresponding author on reasonable request.
